# LIVING WITH DEMENTIA: COMMUNITY-BASED CARE PARTNER EDUCATION EVALUATION RESULTS

**DOI:** 10.1093/geroni/igad104.1661

**Published:** 2023-12-21

**Authors:** Erica Husser, Rollin Wright, Janice Whitaker, Diane Berish, Olivia Rubio, Donna Fick

**Affiliations:** Penn State University, State College, Pennsylvania, United States; Penn State Health at Hershey Medical Center, Hershey, Pennsylvania, United States; Nese College of Nursing, Penn State University, State College, Pennsylvania, United States; The Pennsylvania State University, University Park, Pennsylvania, United States; Penn State University, University Park, Pennsylvania, United States; Penn State University, State College, Pennsylvania, United States

## Abstract

A mixed methods overall series evaluation was administered at session four in each location to assess preparedness, perceived increase in caregiver self-efficacy and wellness, development and implementation of skills, and overall program value. Fifty-five participants (73% response rate) completed the evaluation; 44 (80%) were care partners of a person living with dementia (PLWD). Twenty-four (44%) participants attended all four sessions, 9 (16%) attended three, 8 (15%) attended two, and 11 (20%) attended one. Those who attended all four sessions were more likely to strongly endorse that they felt better prepared (96% vs. 75%; X2 = 3.7, p = 0.05). On a 5-point scale (1 = Strongly disagree, 2 = Disagree, 3 = Neither agree/disagree, 4 = Agree, 5= Strongly agree), 38 (85%) reported agree or strongly agree to improved preparedness to meet the needs of their PLWD, and 41 (93%) reported improved confidence as a care-partner. The top 5 communication skills learned: Using fewer words (89%); Slowing down speech (89%); Focusing on the relationship (86%); Ending an encounter with thank you (86%); and Going with the flow (84%). Content analysis of qualitative data suggest care-partners’ new understanding and improved communication skills were having a positive influence on the pace and quality of daily life and reported less conflict and improved mood; challenges persisted with activities of daily living, such as showering and toileting. Programs like LWD that provide care partners education, skill development and support for self-care can improve daily life for care-dyads living with dementia.

